# Safety Testing: Moving Toward Alternative Methods

**DOI:** 10.1289/ehp.0901704

**Published:** 2010-01

**Authors:** Linda S Birnbaum, William S. Stokes

**Affiliations:** National Institutes of Health, Department of Health and Human Services, Research Triangle Park, North Carolina, E-mail: birnbaumls@niehs.nih.gov; U.S. Public Health Service and Director, NTP Interagency Center for the Evaluation of Alternative Toxicological Methods, NIEHS, National Institutes of Health, Department of Health and Human Services, Research Triangle Park, North Carolina

Protection of workers and consumers demands that safety testing accurately detects chemicals and products that can cause injury or disease. Chemicals make our lives more comfortable, but accidental and improper exposures to chemical substances continue to have a significant public health impact. According to the Institute of Medicine (2004), more than 4 million poisonings occur annually in the United States, and poisonings are the second leading cause of injury-related deaths, exceeded only by automobile accidents. Common household products such as household cleaners cause about 125,000 eye injuries each year. Skin diseases, injuries, and disorders, including contact dermatitis and allergic contact dermatitis from chemicals, are the most common category of occupational illness.

Sixteen years ago the National Institutes of Health (NIH)Revitalization Act (1993) was signed into law with a little-noticed directive to the National Institute of Environmental Health Sciences (NIEHS) to develop a process to achieve regulatory acceptance of scientifically valid alternatives to animal-based safety testing. That directive gave rise to an interagency effort that has become increasingly effective in identifying, evaluating, and validating new test methods, including alternative methods, that reduce, refine, and replace animal testing—commonly referred to as “the 3Rs.”

The NIEHS, along with 14 other U.S. Federal agencies, collectively known as the Interagency Coordinating Committee on the Validation of Alternative Methods (ICCVAM 2009b; ICCVAM Authorization Act of 2000), has made important and substantial progress benefiting both public health and animal welfare.

ICCVAM’s focused efforts have resulted in approved alternative test methods for many types of product safety testing, including the four most commonly conducted safety tests: acute oral toxicity, dermal irritation/corrosion, ocular irritation/corrosion, and allergic contact dermatitis. The first test method evaluated and recommended by ICCVAM was a mechanism-based assay for allergic contact dermatitis testing that uses fewer animals and eliminates pain and distress compared to the traditional assay. The comprehensive ICCVAM evaluation served a key role in achieving rapid international acceptance and widespread use of this alternative method. Since that time, ICCVAM has contributed to the national and/or international regulatory acceptance of 27 alternative safety testing methods, including 17 that do not use live animals (ICCVAM 2009b).

In April 2009, an ICCVAM initiative fostered an international agreement between the United States, Canada, Japan, and the European Union that is expected to further reduce animal use in product toxicity testing worldwide (Hood 2009; ICCVAM 2009a; NIEHS 2009). The agreement involves globally coordinated, high quality validation studies and peer reviews executed using a transparent process that should speed the international adoption of alternative toxicity testing methods.

In the United States, federal law requires that new test methods must be determined to be valid for their proposed regulatory use before their adoption by regulatory agencies (ICCVAM Authorization Act of 2000). The law also stipulates that the new alternative test methods must provide equivalent or improved protection compared to existing methods. ICCVAM’s comprehensive scientific evaluations address these legal requirements, expediting acceptance of new test methods, and fulfilling a vital role in assisting agencies in meeting regulatory acceptance requirements.

Promoting the use of accepted alternative methods has—and will continue to have—a huge positive impact on animal welfare. Thanks to the work done by ICCVAM and other organizations, animals are no longer required for many testing scenarios and the number of animals required for safety tests has been dramatically reduced. Where animals must still be used, many test methods have now been refined to significantly reduce or avoid most pain and distress. Most importantly, ICCVAM’s careful evaluation of the usefulness and limitations of these alternative methods will ensure that their proper use will in fact continue to support equal or better protection of people, animals, and the environment.

Some 50 years ago in a book titled *The Principles of Humane Experimental Technique,* two British scholars named William Russell and Rex Burch described the concept of the 3Rs as a way to advance animal welfare (Russell and Birch 1959). They would undoubtedly be pleased to learn of the progress forged by ICCVAM.

## Figures and Tables

**Figure:**
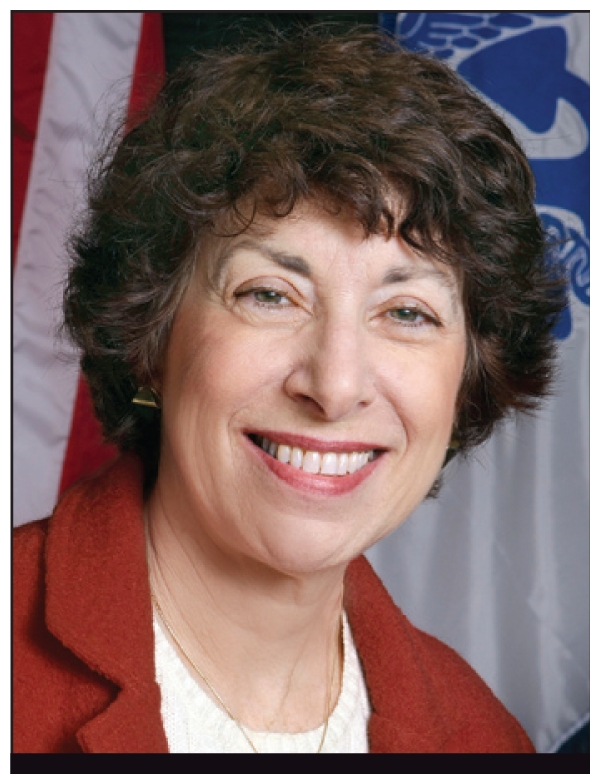
Linda S. Birnbaum

**Figure:**
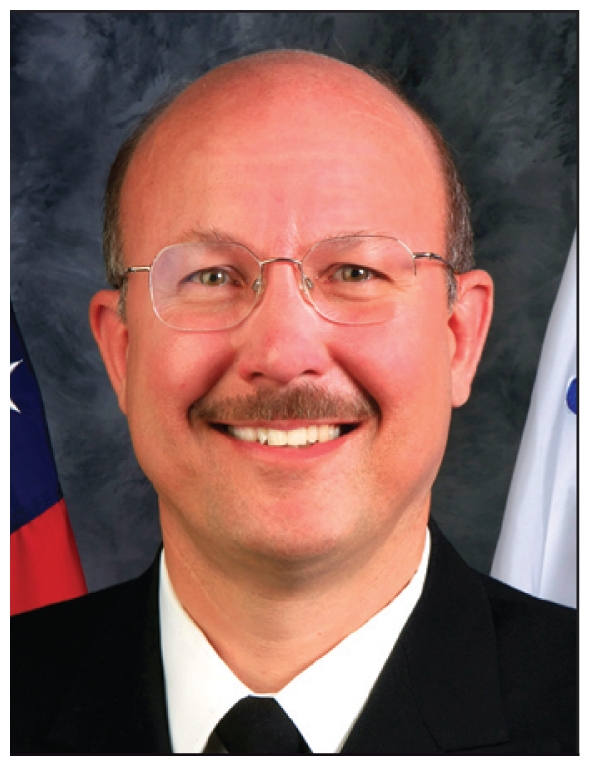
William S. Stokes
